# Integration of patient safety into educational curricula and continuing professional training - comparison of patient safety competency models

**DOI:** 10.3389/frhs.2026.1757601

**Published:** 2026-04-01

**Authors:** Sini Eloranta, Saara Ketola, Tuija Ikonen

**Affiliations:** 1 Department of Nursing Science, University of Turku, Turku, Finland; 2 Turun Ammattikorkeakoulu, Turku, Finland; 3 Osterbottens Valfardsomrade - Pohjanmaan Hyvinvointialue, Vaasa, Finland; 4 Coxa Hospital, Tampere, Finland; 5 Public Health, University of Turku, Turku, Finland

**Keywords:** competency models, continuing professional development, education, healthcare, patient safety

## Abstract

**Introduction:**

Patient safety is a fundamental component of high-quality healthcare, and integrating its principles into health professional education and continuing professional development is essential for ensuring safe care. This article describes and compares four international patient safety competency models from high-income regions (Australia, Canada, the United Kingdom, the Nordic countries), and the WHO Multi-professional Patient Safety Curriculum Guide and analyzes their similarities and differences. In this descriptive comparative review, the objective is to identify which competency domains each model emphasises, and which competencies are critical for advancing patient safety across healthcare systems.

**Methods:**

A thematic analysis of the patient safety competency models was conducted, focusing on their core competency domains and identifying the competencies most critical for advancing patient safety across healthcare systems in high-income regions.

**Findings:**

Common competency domains include safety culture, systems thinking, teamwork, communication, risk management, human factors, and continuous learning. However, there are notable differences: for example, the Nordic framework for knowledge and skills emphasizes technology and preparedness, Australia highlights ethics, and Canada focuses on resourcing.

**Discussion:**

The comparison of four patient safety competency models in high-income countries, and the WHO Multi-professional Patient Safety Curriculum Guide shows that they share a common set of core domains, providing a strong basis for aligning patient safety education in high-income countries. At the same time, country specific emphases highlight the need for contextual adaptation.

## 1 Introduction

Patient safety is universally recognized as a cornerstone of high-quality healthcare, with the prevention of errors and harm constituting a fundamental priority at all stages of care delivery (1). Despite sustained global efforts to improve safety standards, adverse events remain a significant challenge. This underscores the need for proactive strategies that begin at the educational level. Systematically integrating patient safety principles into health professional curricula and continuing professional education is essential to cultivating a workforce capable of delivering safe, effective, and high-quality care (2). Equally important is the systematic evaluation of patient safety education—through assessments of knowledge acquisition, skill development, and application in clinical practice—to determine its effectiveness and guide curriculum refinement.

The Organisation for Economic Co-operation and Development (OECD) and the European Union (EU) emphasize the systematic incorporation of patient safety into educational programs (3, 4). The Multiprofessional Patient Safety Curriculum Guide (5) stresses that patient safety should not be taught as a standalone, isolated module, but rather as a fundamental, recurring element of professional identity formation, clinical reasoning, and organizational culture. Nevertheless, substantial variability persists in how patient safety competencies are taught, internalized, and applied in practice, both during formal education and throughout employment, limiting comparability and undermining efforts to establish shared standards.

The World Health Organization (WHO) has led global action to advance patient safety education by providing internationally recognized patient safety guideline to support teaching in this area. The first WHO Patient Safety Curriculum Guide was published in 2009 for medical schools (6), followed in 2011 by the Multiprofessional Patient Safety Curriculum Guide extending its scope to nursing, pharmacy, midwifery, dentistry, and other professions (5). In parallel, many countries have developed patient safety competency models tailored to local regulation, health system structures, and cultural context. Such countries in high-income settings include at least, Australia (7), Canada (8), the United Kingdom (9), and the Nordic countries (10). The WHO Global Patient Safety Action Plan 2021–2030 (11) sets seven strategic goals that further emphasize integrating patient safety into education at all levels. Patient safety competency resources are critical to achieving these aims by defining competencies, standardizing content, and ensuring quality and comparability across programs. They provide guiding models for systematic curriculum development aligned with international standards and best practices, establishing a common language that facilitates design, implementation, and evaluation, while supporting competency development from basic training through continuing education.

Recent literature across medicine, nursing, pharmacy, and the allied health professions highlights several effective strategies for integrating patient safety into professional education. These include early incorporation of patient safety content, longitudinal integration across multiple courses, and the use of experiential learning methods such as simulation (12, 13). Despite these promising approaches, significant barriers to successful integration persist. Common challenges include overcrowded curricula, limited availability of evidence-based pedagogical methods, insufficient faculty preparedness, inadequate organizational support, and difficulties in measuring educational outcomes, particularly those related to attitudes and safety culture (14–20). Consequently, sustainable integration requires not only pedagogical innovation but also broader organizational development to support and maintain change.

No guidelines exist at worldwide or EU level which address how patient safety should be taught (18). As a result, individual HEIs and universities determine how patient safety should be taught. Recent systematic review reported considerable variation in how patient safety education is implemented at the undergraduate level in nursing education, including differences in course content, curricular placement, evaluation processes, and assessment tools (21). Similar inconsistencies have been documented in earlier studies (22, 23).

Ensuring systematic patient safety education in health care education programmes is important for creating a safer healthcare system. Leveraging established patient safety competence resources may therefore offer educators structured, evidence-based objectives and content as well as assessment tools that can help standardize teaching practices and strengthen patient safety competencies among health professional students (1, 16, 18, 21).

The improvement of safety in healthcare worldwide depends in part on the knowledge, skills and attitudes of staff providing care (17). The WHO Global Report on Patient Safety (24) urges countries to invest in competent development, as safe practices cannot be effectively embedded into everyday work without it.

The present article describes and compares four international patient safety competency models from high-income regions and the WHO Patient Safety Curriculum and analyzes their similarities and differences. The objective is to determine which competency domains for each patient's safety competency models emphasize and to identify the most critical competencies for advancing patient safety across healthcare. This comparison addresses a gap in the existing literature: previous studies have primarily examined single patient safety competency models focused on pedagogical implementation strategies, whereas comprehensive cross model- analyses and their implications for curriculum design remain scarce. The findings of this analysis are intended to inform curriculum planning and health care education, strengthen competency development, and support the harmonization of international and national safety standards.

## 2 Methodology and methods

### 2.1 Selection of frameworks

In this descriptive comparative review, the following patient safety competency models selected for analysis in addition to the WHO Multi-professional Patient Safety Curriculum Guide (5): the Australian National Patient Safety Education Framework (7), the Canadian Safety Competencies (8), the British NHS Patient Safety Syllabus (NHS) (9), and the Nordic Framework for Patient Safety Knowledge and Skills (10). The selection of the patient safety competency models was guided by purposive, informed judgement (25). We selected these models because they are internationally recognized and structurally comparable, enabling a meaningful comparison of how different countries emphasize patient safety competencies. The WHO Patient Safety Curriculum serves as a global foundation also applicable in middle- and low-income settings, while Australia, Canada, the United Kingdom, and the Nordic countries represent national models used to support education and competency development in high-income countries.

### 2.2 Data analysis

The data were analyzed using qualitative, inductive thematic analysis. The chosen analytical method is considered appropriate for examining textual data (25). Inductive thematic analysis is a data-driven approach in which the analysis is grounded in the material itself rather than guided by a predetermined theoretical framework. In this approach, the themes emerging from the data are placed at the center of the analysis. Thematic analysis offers a flexible methodological approach that emphasizes the data itself (26).

The thematic analysis was carried out by three patient safety researchers in 2025. The analysis proceeded through three stages, and all three researchers ensured the integrity of the analytical process. First, the material was systematically documented. The documenting of the competence resources was predetermined. We utilized a structured Excel template, into which the following descriptive details were extracted from each framework: country, year, intended use, domains/competence areas: safety culture, from blame culture to system error, human/environmental factors, system thinking, understanding complexity, teamwork/communication, responsibilities and values, patient and family engagement, communication, learning from errors, never events, risk management, monitoring and evaluating patient safety, continuous improvement, evidence based practice, continuity of care, ethics, infection prevention, medication safety, digital safety, and other notes.

Second, the examination of the material imported into the Excel framework continued by reading it multiple times to gain an overall understanding of the dataset. The analysis progressed by examining the data in relation to the study aim and by making observations and notes on similarities and differences between the models, allowing themes to be derived directly from the material. Third, recurring contents and concepts across the models were grouped and organized into themes. To ensure the accuracy of the analysis, the original material was revisited throughout the process, and the themes were refined accordingly. The thematic analysis results were not verified by individuals outside the research team.

Each patient safety model was also analyzed within the research team using a SWOT analysis. This analysis identified the strengths, weaknesses, opportunities, and threats of each framework. The analysis was based on perceptions and a consensus reached among the research team members, and it was not validated by individuals externally to the team.

## 3 Findings

### Descriptive and comparative analysis of patient safety competency models

This analysis examined the WHO Multi-professional Patient Safety Curriculum Guide (5), and four international patient safety competency models from high-income regions: the Australian National Patient Safety Education Framework (7), the Canadian Safety Competencies (8), the British NHS Patient Safety Syllabus (9), and the Nordic Framework for Patient Safety Knowledge and Skills (10). Table 1 presents the key areas of competence for each model. In the results presented in the following sections, the analysis was conducted on the entire framework.

**Table 1:** Comparison of Patient Safety Competency Models by main competence areas.

Patient Safety Competency Models	Australian National Patient Safety Education Framework (2005)	WHO Multi-professional Patient Safety Curriculum Guide (2011)	Canadian Safety Competencies (2020)	NHS Patient Safety Syllabus (UK) (2022)	Nordic Patient Safety Framework (2024)
Main competence areas	*N* = 7	*N* = 11	*N* = 6	*N* = 5	*N* = 15
Patient safety concepts, definitions, and importance		✓			✓
Systems thinking/awareness		✓		✓	✓
Human factors and environmental factors		✓	✓	✓	✓
Communication, responsibilities, duties, and roles	✓	✓	✓		✓
Teamwork			✓		✓
Learning from errors/incidents, continuing learning	✓	✓		✓	✓
Risk management		✓	✓		✓
Contribute to a culture of patient safety			✓		
Infection prevention and control		✓			
Medication safety	✓	✓			
Patient and family's involvement/engagement Patients and families as co-creators		✓			✓
Safety culture and leadership		✓			✓
Education and continuous improvement	✓	✓			
Being ethical	✓				
Identifying, preventing and managing adverse events and near misses.	✓		✓		✓
Monitoring and evaluating patient safety					✓
Using evidence and information	✓				
Creating safe systems				✓	
Technology, digitalisation and patient safety					✓
Preparedness and contingency planning					✓
Risk areas and special situations					✓
Organizational culture and patient safety					✓
Being sure about safety				✓	

### 3.1 Common domains across patient safety competency models

The patient safety competency models aim to support educational and health care organizations in developing and maintaining patient safety competencies across all levels from basic education to continuing professional development and leadership.

Common domains emphasized across all competency models include a) Patient safety culture and systems thinking, b) Teamwork and communication, c) Risk management and adverse events, d) Human factors and performance, e) Learning from errors and incidents, and f) Evidence-based practice and continuous learning.

System-level thinking and safety culture are reflected in unified procedures to prevent adverse events and in promoting open reporting of deviations. Teamwork and clear communication are essential for interprofessional collaboration and shared reporting tools. Learning from errors and managing adverse events is implemented through root cause analyses and feedback sessions, where lessons learned inform new practices. Human factors are addressed through ergonomic workspace design and training to recognize human error. Evidence-based practice and continuous learning are supported by regularly updating patient safety training based on current research and systematically assessing competencies.

### 3.2 Differences and unique features

Differences between patient safety competency models were also observed. For example, the Australian National Patient Safety Education Framework (7) places strong emphasis on ethics, which is less prominent in other frameworks. The Nordic Framework for Patient Safety Knowledge and Skills (10) introduce new competence areas related to the development of the operating environment, such as the role of technology, which are not as comprehensively covered elsewhere. Furthermore, it connects preparedness and contingency planning to patient safety. Patient involvement from the WHO Multi-professional Patient Safety Curriculum Guide (5) is reproduced only in the Nordic Framework for Patient Safety Knowledge and Skills (10) in the form of patients and families as co-creators.

Country-specific priorities were evident. Australia (7) highlighted workplace behaviors and values, including bullying, alongside lifelong learning, onboarding and supervision for new tasks, professional and ethical conduct, digital health, and equipment safety. Canada (8) focused on interprofessional patient safety competencies, organizational learning, and underscored the critical importance of adequate funding and staffing. The Nordic countries (10) add further elements, such as functional safety, radiation safety, and information security. It dedicates an entire competence area to organizational culture and includes preventive measures for falls, pressure ulcers, bladder overdistension, healthcare-associated infections, and suicide as healthcare-related injury. Additional considerations include work capacity and competence, as well as reflections on the second victim phenomenon and situations where healthcare workers face threats and violence.

### 3.3 SWOT analysis of patient safety competence models

All patient safety competence models are intended for broad applications and are designed to be utilized by healthcare professionals, educators, educational institutions, policymakers, and healthcare administrators. The WHO Multi-professional Patient Safety Curriculum Guide (5) offers a wide set of competence models including teaching materials, sample lectures, and assessment tools for multi-professional training. The Australian National Patient Safety Education Framework (7) provides links to e-learning modules. The Canadian Safety Competencies framework (8) includes simulation-based tools and competency assessment resources. The NHS Patient Safety Syllabus (9) is supported by tiered online courses and practical learning packages. The Nordic framework for Patient Safety Knowledge and Skills (10) offers additional guidance for applying competence areas and integrating them into national training programs. Thus, all models are completed by varying sets of competence resources as online modules, implementation guides, and evaluation tools, that are provided to facilitate adoption and continuous professional development.

A comparative SWOT analysis of five international patient safety competence models revealed both common and distinctive features (Table 2). All models emphasize strong conceptual foundations for patient safety and a clear structure. Differences were particularly evident in the competence models’ currency: the Australian (7) and WHO Multi-professional Patient Safety Curriculum Guide (5) are conceptually robust but require updates to better reflect the current healthcare environment. Except for WHO, all models have been tailored to meet the specific needs of their respective countries and regions. The Nordic framework for Patient Safety Knowledge and Skills (10) is new and tailored to Nordic needs and highlights cross-border collaboration. Combining the strengths of these models, such as the contextual relevance of the Nordic model (10), the competency-based approach of the Canadian framework (8), the digital pedagogy of the NHS syllabus (9), and the global perspective of the WHO framework (5), can enhance patient safety education in a rapidly changing healthcare environment.

**Table 2:** SWOT analysis of patient safety models.

Patient Safety Competency Models	Australian National Patient Safety Education Framework (2005)	WHO Multi-professional Patient Safety Curriculum Guide (2011)	Canadian Safety Competencies (2020)	NHS Patient Safety Syllabus (UK) (2022)	Nordic Patient Safety Framework (2024)
Strengths	Simple, and clear structure. Promotes a unified safety culture and standards across different professional groups.	An internationally recognized framework.	Interprofessional approach, updated in 2020. Clear and practical. Presented concrete learning objectives (skills, knowledge, attitudes) for patient safety.	Clear structure. A new and up-to-date framework that incorporates modern patient safety principles and technologies. Includes practical examples and digital learning solutions, which support broad implementation.	A new and up-to-date framework that incorporates modern patient safety principles and technologies. Latest research.
Weaknesses	Published in 2005, therefore, some of the content may be outdated. Contextuality.	Published in 2011, so some of the content may be outdated in relation to current technologies and practices. Lacks national context.	Less considering changes in the healthcare environment, such as digitalization.	Strongly tied to NHS structures.	New – implementation still in progress.
Opportunities	Updating and integrating with newer models.	It can be utilized globally as a standard for patient safety education. An update could take into account the changes brought by healthcare digitalization and artificial intelligence.	2nd Edition is strengthened by advancements in collective knowledge, including patient and family partnership, leadership, quality improvement and cultural competency concepts.	A modular structure that enables flexible learning for different professional groups. Collaboration with WHO and other global stakeholders could help establish unified standards.	Harmonization across Nordic countries. Collaboration with WHO and the EU could strengthen international positioning.
Threats	Obsolescence. The rapidly changing healthcare environment can render the framework obsolete without continuous updates.	The changing healthcare environment can render the framework obsolete without continuous updates. Cultural differences and challenges in application.	An update could consider the changes brought by healthcare digitalization and artificial intelligence.	A rapidly changing environment requires continuous updates.	Implementation requires significant training resources. Regulatory differences between countries.

## 4 Discussion

Our analysis of the WHO Multi-professional Patient Safety Curriculum Guide (5), and four international patient safety competency models from high-income regions highlights both similarities and differences in competency requirements. Common areas, such as safety culture and systems thinking, teamwork and communication, risk management, human factors, learning from errors, and evidence-based practice, form a shared foundation for patient safety expertise. These competencies provide a strong basis for establishing international standards in patient safety education. Recognizing and integrating these shared areas enables curriculum harmonization across high-income countries and supports the development of national programs aligned with global recommendations, ensuring that healthcare professionals’ skills meet international benchmarks.

Despite this common foundation, notable differences exist. The comparison reveals which competency areas each model emphasizes and how these differences relate to national needs. For example, Australia (7) focuses on workplace behaviour, Canada (8) highlights staffing and funding, and the Nordic framework for Patient Safety Knowledge and Skills (10) introduces competencies for functional safety, information security, and preparedness. The Nordic Framework also reflect the digitalization of healthcare and its implications for safety (10). It appears that international competency standards can rely on shared core domains, but implementation at the regional level requires flexibility and sensitivity to context. National programs, in turn, need to incorporate additional elements that reflect the specific priorities and characteristics of their healthcare systems.

Patient safety competence models differ in their intended audiences and resources. WHO provides comprehensive teaching materials for educators and globally regardless of economic settings (5); Australia and Canada emphasize interprofessional learning (7, 8); the NHS syllabus is tailored for UK staff (9); and the Nordic framework targets professionals and leaders (10). All offer supplementary tools, such as online modules, simulations, and assessments, to support implementation and continuous development.

The analysis of these competence models shows that while all emphasize a strong conceptual foundation and clear structure, their differences offer significant opportunities for development. Variations, such as contextual tailoring, the use of digital pedagogy, and the inclusion of a global perspective, indicate that developing patient safety competencies cannot rely on a single model but require flexibility and diversity. By combining the strengths of different patient safety competence models, it is possible to create an approach that supports both local needs and international collaboration. This strategy is particularly important in a rapidly changing healthcare environment, where continuous competency updates and innovative learning methods are essential.

Understanding the similarities and differences among patient safety competence models is not only important for curriculum design but also for ensuring effective implementation. While competence models provide conceptual foundations and structural guidance, their success in practice is strongly influenced by organizational and cultural conditions. Visible and sustained leadership commitment, alignment with safety strategies, adequate resources, and a supportive, non-punitive culture are critical drivers of impact. Without these, even well-designed patient safety competence resources struggle to achieve lasting change. Therefore, future-oriented patient safety education must combine the strategic insights gained from competence resources analysis with organizational readiness and cultural transformation. This includes embedding patient safety throughout all stages of professional learning from undergraduate education to continuing development supported by clear competency standards, innovative pedagogy, and opportunities for real-world application. In increasingly complex healthcare environments in high-income regions, this integrated approach is essential for improving outcomes, reducing harm, and sustaining a culture of safe, high-quality care.

A limitation of this review is that the four patient safety models apart from WHO Patient Safety Curriculum analysed originate rom high income regions. As such, the findings may over-represent the challenges and demands of high-income settings rather than a globally representative set of competency resources. This limits the geographic generalisability of the findings, and the results should not be assumed to represent global competency expectations. In future research, it will be important to evaluate patient safety competence models from the perspective of low and middle-income countries, to identify how local conditions, resource constraints, cultural practices, and healthcare system structures influence patient safety competence and education.

## 5 Conclusion and implications for education

The comparison of the WHO Multi-professional Patient Safety Curriculum Guide (5), and four international patient safety competency models from high-income regions shows that they share a common set of core domains, providing a strong basis for aligning patient safety. At the same time, countryspecific emphases highlight the need for contextual adaptation.

Based on the findings of this study, in the short term, educational programs should ensure that the internationally shared core competencies become a clear and structured part of teaching. Based on the analysis, these key competency areas, patient safety culture, systems thinking, teamwork and communication, risk management, human factors, and learning from errors appear across all examined patient safety competence models and provide an immediate basis for harmonizing education.

In the long term, it is recommended that national patient safety competencies be developed in a way that integrates the strengths identified in the analysis, for example the emphasis on technology in the Nordic Framework and ethics and workplace safety in the Australian Framework. Leveraging these different national emphases enables competence development that is internationally comparable yet locally relevant. Ensuring that these competence resources remain sufficiently practical and applicable is crucial, as this enables educators and healthcare organizations to effectively strengthen patient safety competencies in real world settings.
